# Occupational Health: Lunar Lung Disease

**DOI:** 10.1289/ehp.116-a423a

**Published:** 2008-10

**Authors:** Adrian Burton

With the National Aeronautics and Space Administration (NASA) finalizing plans to begin construction on a manned lunar outpost by 2020, the sun is rising on a whole new world of environmental and occupational health. Could longer space missions, reduced gravity, and moondust be a dangerous combination for lunar astronauts’ pulmonary health? Results published in the August 2008 issue of the *European Journal of Applied Physiology* suggest so.

“Moondust has properties similar to silica, a mineral commonly encountered in mining operations known to cause silicosis and other lung problems,” explains principal investigator Kim Prisk, a researcher with the Human Factors and Performance Team at the National Space Biomedical Research Institute. “To be safe we need to know how much dust can get into astronauts’ airways, where it deposits, how long it stays, and just how toxic it might be.” Such information is essential to mission designers who must know what air purification equipment to haul moonward and how extensive post-moonwalk decontamination procedures must be in order to clean up the clingy dust.

To determine how much and where moondust might deposit in the lungs, Prisk’s team performed experiments under lunar gravity conditions during parabolic flights aboard NASA’s Microgravity Research Aircraft. Up to six subjects breathed in aerosols (104 particles/mL) of 0.5- or 1-μm polystyrene latex beads (representative of moondust particle sizes) at a constant rate through a mouthpiece. The number of beads entering and leaving the subjects’ breath was measured using a photometer, which revealed how much “dust” remained in subjects’ airways. Compared with terrestrial conditions, lunar gravity reduced the total quantity of 0.5-μm beads settling in the airways by 25% and 1-μm beads by 32%, explains first author Chantal Darquenne, an associate professor in the Department of Medicine at the University of California, San Diego. But while this may seem like good news, further experiments showed that the beads that settled were more likely to do so in the alveoli, clearance from which may be difficult. Whereas particles would be deposited in the upper airways under terrestrial conditions, under lunar conditions they would remain available to be eventually transported deeper into the lung.

“NASA is learning that [different] moondusts have toxicologically worrisome properties including reactive surfaces, large surface areas per unit mass, a high content of particles greater than 0.1 μm, and metallic iron impregnation,” says John James, chief toxicologist at the NASA Johnson Space Center in Houston, Texas. “We must be careful not to suppose that there is one level of toxicity for lunar dust,” he adds. “It is likely that there is considerable variability in the size distribution and mineral composition of dust from one location to another. This could mean several-fold variability in the inherent toxicity of the dusts.”

“Occupational dust diseases can take decades to develop among exposed workers on Earth, but if the alveoli are more affected on the moon and clearance is reduced, disease could develop more quickly in astronauts—especially if lunar dust proves very toxic,” says David Goldsmith, an associate research professor of environmental and occupational health at The George Washington University in Washington, DC. Emphasizing that we should recognize moondust-exposed astronauts as dusty trades workers in space, he points out that chronic silica exposure can also lead to lung cancer, kidney ailments, and autoimmune diseases in occupationally exposed workers on Earth. He adds, “In space and on Earth these conditions are fully preventable with proper respiratory protection including engineering controls.”

This high-flying research may also have a down-to-Earth application. By determining where particles of different size settle in the airways under terrestrial gravity conditions, it may be possible to learn how to deliver drugs accurately to different areas of the lungs.

## Figures and Tables

**Figure:**
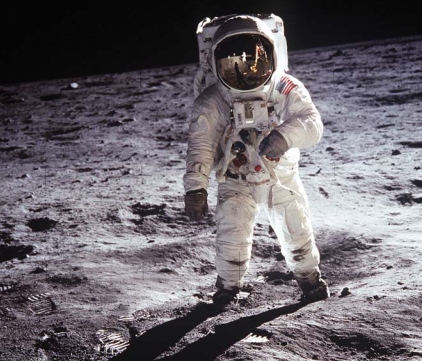
Moondust has properties similar to the dust that causes lung disease in Earthbound miners.

